# The factors that affect members’ use of a beauty industry matchmaking platform: Validation of the COM-B extended model

**DOI:** 10.3389/fpsyg.2022.976109

**Published:** 2022-10-10

**Authors:** Yang-Wen Chang, Yen Hsu

**Affiliations:** The Graduate Institute of Design Science, Tatung University, Taipei, Taiwan

**Keywords:** COVID-19, COM-B, fashion sense, beauty industry matchmaking platform, behavioral change

## Abstract

The global impact of COVID-19 has seriously affected health and livelihood in every country or region, especially in terms of physical consumption behaviors. Hairdressing is an essential physical consumption behavior. To prevent infection, the consumption model for using the beauty industry matchmaking platform (BIMP) has been used during the pandemic. This study investigates the changes in the behavior of media app users in the beauty industry in the post-epidemic era of COVID-19. The COM-B model is the basis for a research framework to study the factors that affect changes in behavior in the areas of Capability, Motivation, and Opportunity of the theoretical framework. A new dimension of fashion sense has expanded the application and validation of the COM-B model to determine the causal relationship between the ability to pursue beauty, motivation, fashion sense, and opportunities by using the platform and the dimension of user behavior. The study finds that fashion sense in the BIMP has a positive and significant impact on beauty care ability, self-motivation to pursue beauty and future cooperation opportunities. The ability, motivation and opportunity to act are all positively significant, which is in agreement with the theoretical framework of the COM-B model. There is no mediating effect for motivation between fashion sense and behavior. The results of this study show that increasing the sense of fashion for members using the BIMP will increases active behavior for members using the platform. This study also proposes practical suggestions for the operation of the BIMP based on the results.

## Introduction

The COVID-19 pandemic means that governments around the world must administer an effective vaccine and create herd immunization (Shahcheraghi et al., 2021). Infections that are caused by the effects of virus mutations continue to threaten health and threaten an economic and social crisis (Clemente-Suárez et al., 2020; Mishra, 2020; Shereen et al., 2020). Due to the significant impact on the livelihood economy Mehta et al. (2020), determined whether new consumer behavior in the light of COVID-19 is permanent or temporary. The study showed that consumers also experience a behavioral change due to economic instability. This change includes learning from the crisis, changing needs and personalities, developing new cultures, and new market segments and developing new consumer behavior models in an attempt to improve the economic environment.

Social networking is the main tool for the dissemination of information in modern society (Westerman et al., 2014). BIMPs that are closely related to the use of demand are also an important element of the daily life of users (Sun et al., 2013). In the COVID-19 environment, individuals may also use social media to adjust their consumption by talking to friends or sending messages, especially during periods of uncertainty and panic (Makridis and Wang, 2020).

In the post-epidemic era, the contactless economy has enabled Taiwan’s e-commerce channels to maintain double-digit growth. A market statistics report, Kantar, shows that real consumption for e-commerce shopping in the beauty industry in the first half of 2020 has grown by more than 30% for Shopee, Momo Fubon Shopping, and Facebook group shopping, mainly due to online shopping.

Online shopping was mostly dominated by young people but the epidemic has increased online shopping across all age groups. Official websites, e-commerce media platforms, social shopping, reviews, trial use, drugstore promotions and other consumer data shows that there is an opportunity to incorporate a new wave of customers after the epidemic. Consumers seek more positive behaviors to embrace the new normal of consumer habits (Kantar, 2020). Gerstell et al. (2020) found that optimism and new attitudes toward consumer behavior have been fostered by the epidemic and there is renewed concern about personal, household safety and overall public health.

People become more confident because of grooming and dressing to achieve a good appearance (Linardon et al., 2019). Outings are less frequent during a pandemic but the beauty function is clearly self-healing as there is little opportunity to wear makeup and relieve mental anxiety (Pikoos et al., 2020). However, due to changes in consumption habits and consumption patterns to avoid infection from the virus, many beauty consumption channels have shifted from offline to online appointments or in-home services (Sheth, 2020). Consumers who seldom used APPs in the past started to use them for consumer activities (Watanabe and Omori, 2020). Gerstell et al. (2020) showed that online commerce sales are growing and consumers are relying more on online platforms to shop after the pandemic. This change in consumption patterns means that beauty industry players must optimize digital campaigns in order to maintain communication with consumers.

COM-B model is a research framework that was proposed by Michie et al. (2013). It provides an intuitive and pragmatic theoretical framework to explain the factors that influence human behavior, in addition to the traditional theories of rational behavior, planning behavior and technology acceptance patterns. The main factors that influence human behavior are explained in terms of capability, motivation, and opportunity. Depending on the research context, previous studies used 14 domains and 84 dimensions to determine appropriate factors and theories regarding Michie et al. (2011). A study by van der Kleij et al. (2020) that was based on motivational and behavioral models to prevent financial data leakage in organizations found that only capabilities and behaviors are uniquely related and an opportunity and motivation do not affect each other.

Michie et al. (2011) showed that ability are highly correlated, but opportunity and motivation are less correlated because the behavior is different. Atkins and Michie (2013) studied eating habits and behavioral science to show that the COM-B model can be used for many behaviors. Individuals must have the mental capacity, motivation and social opportunities to encourage competitive behavior. Behavioral science can be used to design effective methods to encourage dietary change. COM-B is a flexible theoretical framework that provides the basis for a variety of influences for behavioral change. It is applicable to a variety of fields to verify the factors that influence behavioral change.

Participants in this study are those who use the platform only when they have actual needs, so the motives of the members are clear. Users understand that the system is a new communication channel in response to the changing environment. Despite the spread of COVID-19, human instinct drives the pursuit of beauty. The COM-B model is an appropriate framework for this study to examine the behavioral change of users in the beauty industry matchmaking. According to the characteristics of the BIMP users, adding a sense of fashion to the new structure produces a richer application context.

The COM-B correction model for this study is used to determine: 1.Which factors determine the behavior of BIMP users; 2.Whether user’s sense of fashion is influenced by ability, motivation, and opportunity, and 3.To expand the field of COM-B model research by using fashion sense as a new construct.

## Literature review

### COM-B theory

**Figure 1:**
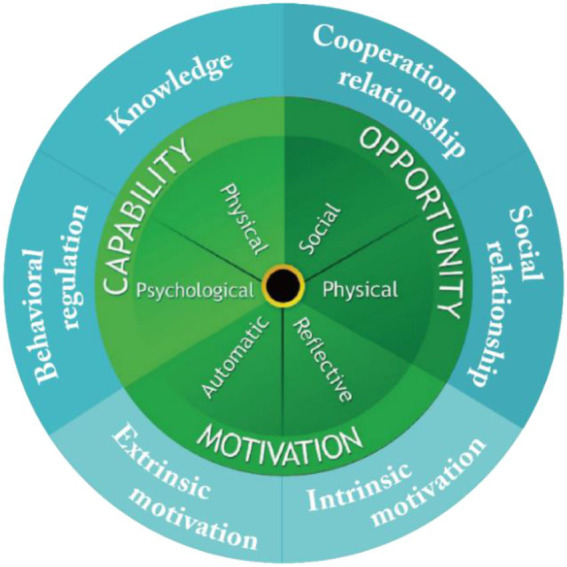
Map of the theoretical domains framework (TDF) to sources of behavior on the COM-B system.

COM-B is a research framework for explaining human behavior that was proposed by Michie et al. (2011) (Figure 1). COM-B does not invent new constructs: it uses the existing Theoretical Domains Framework (TDF), which uses the three main domains of competence, motivation and opportunity, and proposes a guiding framework as the theoretical basis for the corresponding constructs to determine behaviors according to research context (Howlett et al., 2019). The framework is flexible and not too restrictive.

There are many related behavioral theoretical models, such as the theory of rational behavior (TRA) (Ajzen and Fishbein, 1975), the theory of planned behavior (TPB) (Ajzen, 1985, 1991), the technology acceptance model (TAM) (Davis et al., 1989), and the unified technology acceptance model (UTAUT) (Venkatesh et al., 2003). These common models use the construct of psychological attributes to predict changes in attitudes, intentions, subjective regulation, knowledge, perceptual-behavioral control, usefulness, and ease of use or behavior (Taylor et al., 2006).

Cane et al. (2012) noted that the COM-B theoretical framework and the TDF can be used combined. The TDF has 14 domains, which are assigned as elements of the COM-B model and are the elements of the extended construct (Michie et al., 2014). The COM-B model is suited to a variety of contexts and can include groups, populations or corporate sectors.

Kropf et al. (2020) used the Capacity, Opportunity and Motivation Behavior model in the COM-B model to study agrobiodiversity and critical services and threats to ecosystems and showed that farmers’ motivation and related behaviors are influenced by interpersonal and intrapersonal factors. The study shows the comprehensiveness of the COM-B model. The determinants for weekly sedentary time were also determined using the COM-B model to verify its predictive validity. The theory of planned behavior was used to explain which is better. The study showed that the COM-B model has a higher explanatory power (Howlett et al., 2021).

Using the COM-B research framework, many studies develop different constructs in the context of different research domains to extend the theoretical domain framework and use these to predict patterns of behavior. Other variables can also be added to act as antecedents, intermediaries, or mediators. In a study of community pharmacists’ comfort dispensing behavior at a health science center in Texas, United States, the COM-B model of capabilities, opportunities, motivation and behavior was explains 78% of medication changes using a behavioral change and theoretical framework to supplement education for staff functions in pharmacies (Varisco et al., 2020). To study youth developmental sports Preston et al. (2020)), used the COM-B model to determine how coaches’ capabilities, opportunities and motivations influence player behavior. In the United Kingdom, the behavioral outcomes of rent paid by tenants under government housing subsidies were studied using COM-B’s behavioral conceptual framework, in order to formulate behavioral opportunities and motivations in terms of the interaction between behavioral outcomes and subjective capabilities (Hickman and Preece, 2019).

**Figure 2:**
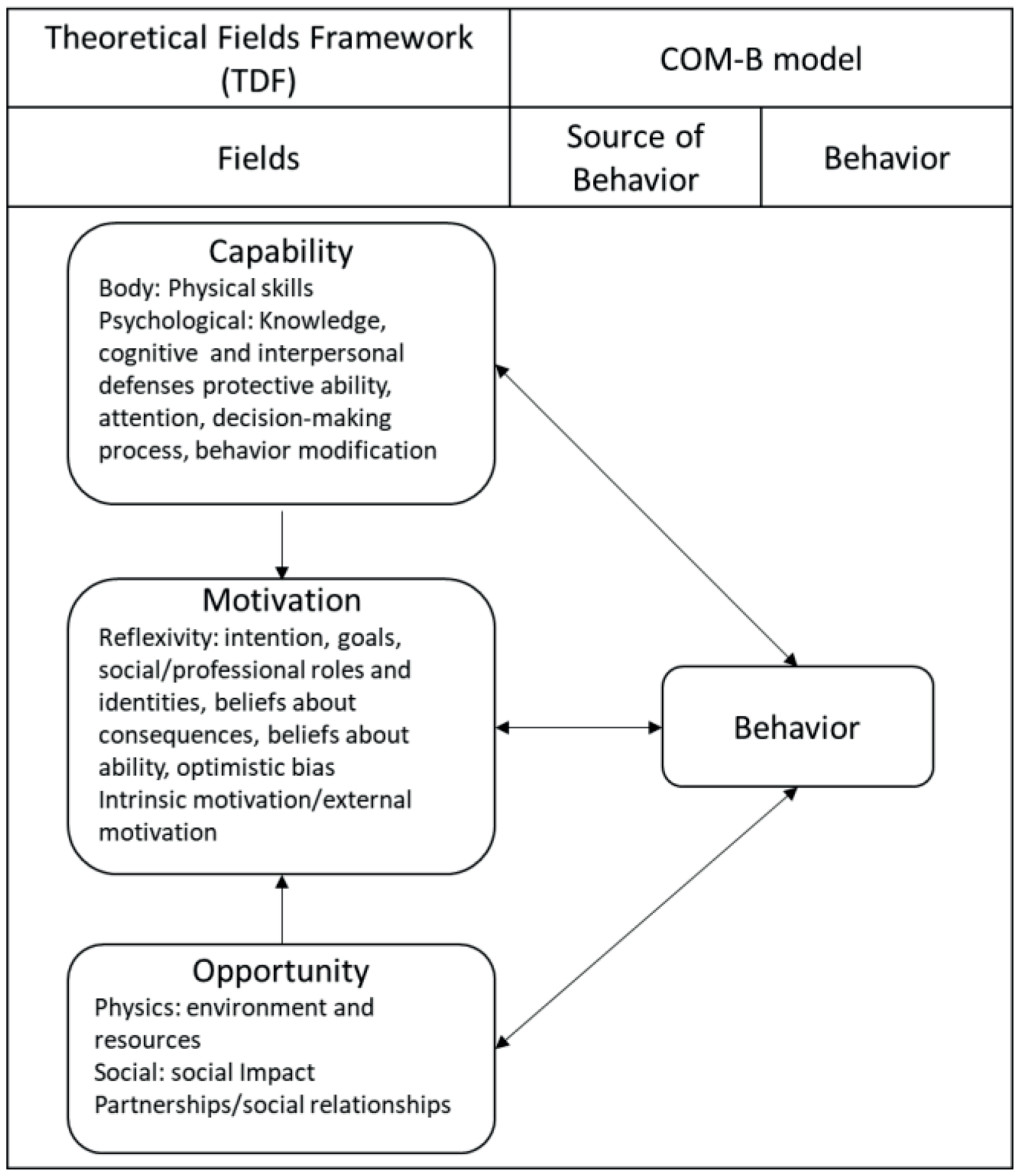
COM-B theoretical fields and models.

Human behavioral development is a decisive factor in the transmission and infection rate for COVID-19. Behavioral science states that the mechanisms that motivate people to behave intrinsically influence the spread of the epidemic (West et al., 2020). This behavioral taxonomy for health-related psychological techniques uses may theories to explain behavioral change (Michie et al., 2011). The COM-B model requires that individuals must have sufficient mental and self-capability (strength, knowledge, and skills), social opportunities (time, social platforms) and motivation (Intrinsic motivation, extrinsic motivation (Michie et al., 2011) (Figure 2).

This study uses the COM-B research framework and proposes a modified COM-B model. Capability, motivation and opportunity are used to determine the factors that affect consumer behavior in the beauty industry in the context of the COVID-19 epidemic. Using the research framework of COM-B, this study determines the factors that influence user behavior in the BIMP, which are capability, motivation, and opportunity. This study proposes that the capability sub-construct is knowledge and behavior regulation, the motivation sub-construct is intrinsic and extrinsic motivation and the opportunity sub-construct contains two sub-structures: cooperative relationships and social relationships (Figure 1).

### Fashion sense

Fashion sense is the communication of popular aesthetics in a specific time and environment, including popular culture and fashionable clothing, or the method by which lifestyle is communicated (Kawamura, 2018; Kaiser and Green, 2021). Regardless of the degree of communication, this is an effect of fame (Bond and Drogos, 2014). Current tools for communication nowadays include various media for entertainment, pleasure and celebrity effect (Oliver et al., 2017).

Famous people adjust their relationships with the media because of a need for fame or a need for an ego support. Celebrities often add psychological motives to the media to create room for discussion and the recognition of being liked (Caughey, 1984). Greenwood et al. (2013) also noted that being recognized in public and having a greater sense of belonging and self-obsession in social networks can cause more online interactions and the effect of being noticed. Recognition brings a sense of cult that is indicative of the role that has become the product of modern social communication media (Giles, 2000). In order to prove the strength and self-confidence of the self, admirers promote self-imitation behavior, in order to have a virtual association and fascination with the celebrity (Friedman et al., 2000).

Entertainment has a subliminal effect on an individual’s perception of life. The effect can be socially beneficial and on some level, entertainment highlights core values, increases human intimacy and has a wide range of positive effects (Oliver et al., 2017). The emotional experience of entertainment involves both positive and negative perceptions so these short-lived pleasures, which are consciously expressed through contact, are appreciated and last longer (Vorderer, 2011).

Media theory states that an empirical assessment of the effect of humanistic entertainment involves mostly self-generated reflections that provoke core values for self-involvement in feeling, autonomy and the purpose of life (Wirth et al., 2012). Recent studies have involved the application of fashion sense. The theoretical discussion treats fashion design as a social concept that is independent of design science and constructive aesthetics and develops and validates relevant principles of art and design (Thornquist, 2014). These studies show that research strategy, sampling strategy and participant rationale are the most common research themes for consumer behavior to determine consumer involvement in the purchase of fashionable clothing. Artificial intelligence has been used for studies of fashion sense for business and consumer applications (Giri et al., 2019).

### Hypotheses development

#### Fashion sense and capability

This study proposes that fashion sense is a cultural phenomenon. It can be regarded as a belief in the pursuit of beauty, and it can influence personal behavior in the pursuit of beauty. This study uses the COM-B model to construct the influencing behavior construct. COM-B competence is based on knowledge and behavioral regulation, to know about satisfying beauty and is considered to have reasonable behavioral regulation due to the expectation factor of always seeking improvement (Grant, 2015). During the COVID-19 pandemic, a study found that perceived behavioral control of the epidemic and the perceived severity of the epidemic at each stage has an impact on behavioral protection and actual switching behavior (Youn et al., 2021). This study proposes hypothesis one:

*H1*: Beauty platform users' fashion sense positively influences beauty care capability.

#### Fashion sense and motivation

Information technology is used to achieve f life and work tasks or to achieve internal satisfaction. Therefore, for beauty platform users, the effect of fashion and entertainment on their lives affects their intrinsic motivation to pursue beauty (Davis et al., 1992). The UTAUT model is the so-called motivation for technology user in anticipation of improvements in personal life and work performance (Davis et al., 1989; Venkatesh et al., 2003).

This study uses the motivational constructs of the COM-B model to explain the behavioral motivations for seeking fashion and beauty. Motivation to pursue beauty is influenced by fashion perceptions, so the greater the fashion perceptions, the greater is the internal and external motivation to pursue beauty, gain self-confidence, and improve interpersonal relationships. This study proposes hypothesis two:

*H2*: Beauty platform users' fashion sense positively influences the motivation for self-seeking beauty.

#### Fashion sense and opportunity

Higgins (2018) in Drapers Magazine suggests that the sales, operational, and customer service demands that are a consequence of fashion sense must be accompanied by rich collaborations and social relationships. The fashion operating model is a challenging market. From a customer-centric perspective, marketing opportunities are inherently social and can only be realized through interaction. This study uses the opportunity construct of the COM-B model to explain how users determine potential opportunities and social relationships in various user environments through this platform. Each user can find opportunities for collaborative and social relationships. This study proposes hypothesis 3:

*H3*: Beauty platform users' fashion sense positively influences opportunities for future collaboration.

#### COM-B capabilities, motivations, opportunities, behaviors

The COM-B model is a behavior modification research framework that is cross-validated with the established TDF. This model framework allows specific individuals to participate in specific activities to produce specific behaviors (B). Each individual has a capacity (C) and the opportunity (O) to perform specific actions in terms of social and personal capacity. Motivation (M) is the psychological drive for behavior and includes internal and external motivations, such as impulsive and rethinking processes and habitual and intentional behavioral states (Cane et al., 2012).

This study determines the behavior of APP users on the BIMP in terms of ability, opportunity and motivation interactions. The capability, opportunity and motivation of member users are used to explain users’ behavior (Stanton et al., 2005). This study proposes the following hypotheses:

*H4*: Beauty platform users' beauty care capability positively influences their motivation to pursue beauty.

*H5*: The opportunity for future collaboration positively influences motivation to pursue beauty using the beauty platform.

*H6*: Beauty platform users' beauty care capabilities positively influence members' behavior towards the beauty platform.

*H7*: Beauty platform users' self-motivation to pursue beauty positively influences members' behavior.

*H8*: The opportunity for future cooperation after the use of the beauty platform positively influences the member's platform usage behavior.

**Figure 3:**
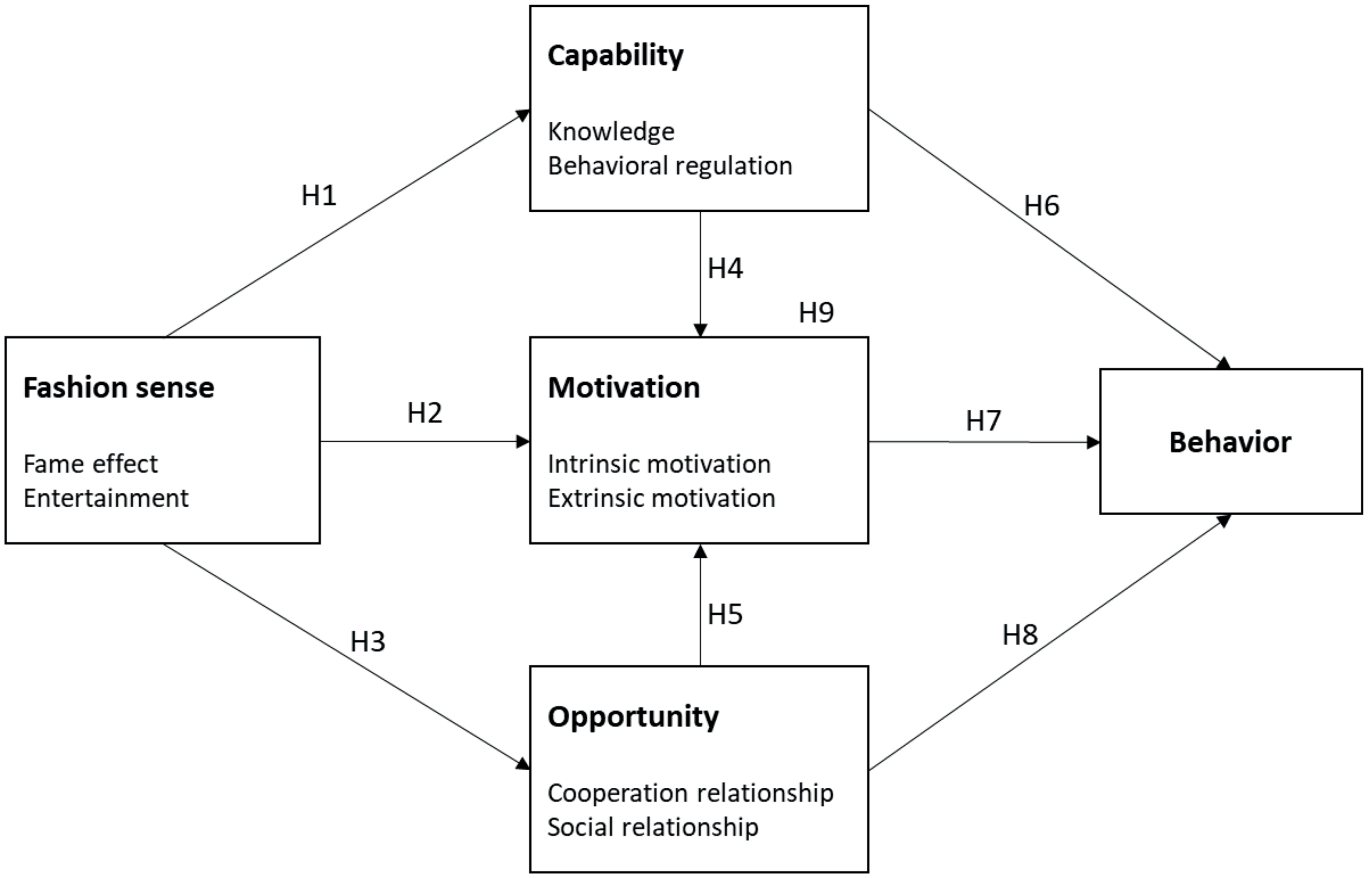
Revised COM-B model.

In terms of the literature review and hypotheses, this study extends the COM-B model by adding a fashion sense construct to validate the various behavioral patterns of BIMP members (Figure 3).

## Research methodology

### Subjects and sampling

The sample for this study is from a three-party appointment service platform of a BIMP, which was supported by the COVID-19 Small Business Innovation Research Program of the Department of Small and Medium Enterprises, Ministry of Economic Affairs, Taiwan. This platform integrates the beauty industry market with the business model with consumers, beauty technicians and beauty store managers, who use mobile app for mutual benefit. At present, the platform is in the formal operation stage. The recruitment of members began in November 2020 and there are currently more than 3,000 members. Using a confidence level of 95% and a confidence interval of 5%, the sample size calculator of The Survey System calculates that a sample size of 341 is required. Members completed a questionnaire voluntarily using a convenience sampling method, after they had used the functions of the APP. A total of 412 questionnaires from members were completed and returned in the period, July–August 2021.

### Design of measurement items

The behavioral constructs for this study are based on the questions in the study by Hsieh et al. (2017), which used the Protection Motivation Theory (PMT) model to study health profile user behavior. Wang et al. (2016) also used the UTAUT model to determine the sharing behavior of social networking site users and constructed questions to evaluate user behavior and to modify the design of the five questions, such as “I always go to the beauty platform” and “I spend a lot of time on the beauty platform.” In terms of the questions pertaining to the capability construct in the questionnaire, the knowledge core questions refer to the study by Abd Rahman et al. (2015), which determined consumers’ knowledge, religion, attitudes and intentions toward halal cosmetics. The research questions were modified by two questions and the core behavioral modification questions were from the study by James et al. (2019).

In the study of the application of the results of the exercise method to influence fitness technology, the research questions were modified to compile three questions, a total of five questions, such as “I understand the basic beauty care and hygiene knowledge” and “If I do not carry out protective measures my friends will give me pressure” and so on. Furthermore, this study includes both intrinsic and extrinsic motivational questions in the motivational construct. The questionnaire questions were adapted from Ma et al. (2018) study on teacher-student relationships and language achievement using a mediated relationship of motivation to effect, and five questions were developed. For example, “I feel confident after my beauty treatment” and “I can improve my interpersonal relationship after my beauty treatment.”

In addition, this study includes partnership and social relationship questions in the opportunity construct. Referring to Thornton et al. (2015), five questions were adapted from the volume scale of the empirical study on internet-oriented behavior. For example, “I can improve my reputation through beauty platforms” and “I can build partnerships through beauty platforms.”

Finally, this study included the fame effect and entertainment effect questions in the fashion sense construct, referring to McCutcheon et al. (2002) research questionnaire on “conceptualization and measurement of celebrity worship,” and modified five questions, such as “I would refer to celebrities’ fashionable outfits” and “I feel happy after going out with reference to celebrities’ outfits.” This study used a seven-point Likert scale to measure the research variable, where (1) represents strong disagreement and (7) represents strong agreement.

## Results

### Analysis of descriptive statistics

The data analysis for this study uses structural equation modeling to verify the influencing relationship for the research hypotheses. D descriptive statistics are used to present the background information for the respondents and to analyze the different data distributions of demographic variables. The measurement model was validated to verify the reliability of the questions and the constructs of the scale using validity factor analysis and discriminant validity analysis. The structural model is verified using path analysis and mediated performance validation. This study uses SPSS 24.0 and AMOS 24.0 statistical software to analyze the data.

The largest group of 109 people are office workers and account for 26.5% of all respondents. The largest group has an income of $30,001–50,000 (157), accounting for 38.1%. 275 respondents are married, accounting for 66.7%. 26.4% of respondents have an education level of college/university. The largest group for the age demographic is 31–40 years old, accounting for 138 people or 33.5%, as shown as Table 1.

**Table 1:** Distribution of demographic variables.

Variable	Label	Frequency	%	Variable	Label	Frequency	%
Occupation	Office worker	109	26.5	Marriage	Married	275	66.7
	Freelance	52	12.6		Unmarried	137	33.3
	Dealer	39	9.5	Education level	High School or below	93	22.6
	Others	68	16.5		College/University	264	64.1
	Service industry	104	25.2		Master or above	55	13.3
	Government employee	33	8.0	Age	Under 20 years old	5	1.2
	Student	7	1.7		21–30 years old	32	7.8
Income (in NTD)	Under 30,000	89	21.6		31–40 years old	138	33.5
	30,001–50,000	157	38.1		41–50 years old	135	32.8
	50,001–100,000	128	31.1		Over 50 years old	102	24.8
	100,00 or more	38	9.2				

The sample for this study includes consumers, technicians and stores. The consumers who are office workers, government employees, students and others account for a total of 52.7% of the sample. The technicians and stores include freelancers, dealers and the service industry and account for a total of 48.3% of the sample.

In this study, the skewness value between-2.060 and − 0.320 and the kurtosis value between-1.069 and 4.611 meet the criteria for an absolute value for skewness of less than 2 and an absolute value for kurtosis of less than 7 that was defined by Kline (2005), as shown in Table 2.

**Table 2:** Analysis of descriptive statistics.

Construct	Item	Mean	Standard deviation	Skewness	Kurtosis
Capability	CAP1	4.70	1.680	−0.582	−0.341
	CAP2	4.50	1.675	−0.528	−0.392
	CAP3	4.30	1.802	−0.367	−0.830
	CAP4	4.33	1.862	−0.409	−0.932
	CAP5	4.14	1.926	−0.320	−1.069
	Average	4.39			
Motivation	MOT1	5.71	1.225	−1.279	2.084
	MOT2	5.64	1.272	−1.312	2.176
	MOT3	6.21	1.243	−2.060	4.336
	MOT4	6.20	1.266	−1.969	3.832
	MOT5	5.87	1.368	−1.384	1.631
	MOT1	5.93			
Behavior	BEH1	5.85	1.254	−1.296	1.870
	BEH2	6.03	1.216	−1.714	3.549
	BEH3	6.06	1.230	−1.826	3.928
	BEH4	6.14	1.137	−1.892	4.611
	BEH5	5.93	1.263	−1.523	2.510
	Average	6.00			
Opportunity	OPP1	5.23	1.450	−0.903	0.612
	OPP2	5.58	1.322	−1.150	1.514
	OPP3	5.57	1.321	−1.108	1.273
	OPP4	5.63	1.300	−1.291	1.835
	OPP5	5.72	1.265	−1.235	1.731
	Average	5.55			
Fashion	FAS1	5.56	1.438	−1.154	1.020
	FAS2	5.64	1.362	−1.221	1.382
	FAS3	5.56	1.404	−1.213	1.466
	FAS4	5.31	1.459	−0.990	0.787
	FAS5	5.33	1.478	−0.958	0.679
	Average	5.48			

### Convergent validity

As shown in Table 4, the standardized factor loadings range from 0.778 to 0.928, which is an acceptable range. This indicates that each question is reliable. The construct reliability for the study constructs ranges from 0.926 to 0.956, which exceeds the value of 0.7 that meets the criteria for other studies. This indicates that each construct has good internal consistency. The final average variance extracted (AVE) ranges from 0.716 to 0.812, which exceeds the 0.5 threshold that is proposed in the study by Hair et al. (2019) shown as Table 3.

**Table 3:** Analysis of measurement model results.

Construct	Item	Z-value	Value of p	STD	SMC	CR	AVE
Behavior	BEH1			0.880	0.774	0.956	0.812
	BEH2	26.553	0.000	0.887	0.787		
	BEH3	28.757	0.000	0.924	0.854		
	BEH4	27.067	0.000	0.909	0.826		
	BEH5	26.836	0.000	0.906	0.821		
Capability	CAP1			0.896	0.803	0.926	0.716
	CAP2	25.755	0.000	0.864	0.746		
	CAP3	20.801	0.000	0.848	0.719		
	CAP4	20.379	0.000	0.841	0.707		
	CAP5	18.404	0.000	0.778	0.605		
Motivation	MOT1			0.874	0.764	0.947	0.782
	MOT2	28.648	0.000	0.925	0.856		
	MOT3	26.933	0.000	0.911	0.830		
	MOT4	25.415	0.000	0.892	0.796		
	MOT5	21.536	0.000	0.816	0.666		
Opportunity	OPP1			0.783	0.613	0.950	0.791
	OPP2	21.157	0.000	0.898	0.806		
	OPP3	22.157	0.000	0.928	0.861		
	OPP4	21.574	0.000	0.916	0.839		
	OPP5	21.528	0.000	0.914	0.835		
Fashion	FAS1			0.894	0.799	0.949	0.788
	FAS2	29.100	0.000	0.909	0.826		
	FAS3	25.691	0.000	0.872	0.760		
	FAS4	27.189	0.000	0.895	0.801		
	FAS5			0.869	0.755		

UNSTD, unstandardized factor loading; STD, standardized factor loading; SMC, square multiple correlation; CR, composite reliability; AVE, average variance extracted.

**Table 4:** Discriminant validity.

Construct	Average variance extracted	Behavior	Capability	Motivation	Opportunities	Fashion sense
Behavior	0.812	0.901				
Capability	0.716	0.415	0.846			
Motivation	0.782	0.477	0.607	0.884		
Opportunity	0.791	0.560	0.427	0.598	0.889	
Fashion sense	0.788	0.476	0.650	0.761	0.658	0.888

The items on the diagonal in bold font represent the square roots of the AVE; off-diagonal elements are the correlation estimates.

### Discriminant validity

In terms of discriminant validity, this study uses the more rigorous AVE method. Previous studies show that discriminant validity must consider the convergent validity for the constructs (Fornell and Larcker, 1981). Therefore, the square root of AVE for each construct must be greater than the correlation coefficient between that construct and other constructs. If this condition is fulfilled, the model has discriminant validity. As shown in Table 4, the square root of the AVE for each component of the diagonal is greater than the correlation coefficient outside the diagonal, so each component for this study has good discriminant validity.

### Model fit

The SEM sample is greater than 200 so the cardinality value is too large. This results in a poor model fit. So the fit value ise corrected using the Bootstrap method (Bollen and Stine, 1992). The results of a comparison with the Bollen-Stine Bootstrap modified model fit, including the fit for the measurement and structural models are shown in Table 5. After fitting with the Bollen-Stine Bootstrap modified model, the fit indices for this study are acceptable.

**Table 5:** Fit Indices.

Fit Index	Acceptable range	Measurement model	Structural model
Chi-square		394.334	400.359
Degree of freedom		265	267
CFI	>0.90	0.989	0.989
RMSEA	<0.08	0.034	0.035
TLI	>0.90	0.988	0.987
GFI	>0.90	0.967	0.967
NFI	>0.90	0.967	0.967
χ2/df	<3	1.488	1.499
AGFI	>0.80	0.958	0.957

### Path analysis

Table 6 shows the results for the path coefficients. These results show that Capability (b = 0.208, p = 0.011), Motivation (b = 0.181, p = 0.032) and Opportunity (b = 0.530, p < 0.001) significantly affect Behavior. Fashion sense (b = 0.554, p < 0.001) significantly affects Capability. Capability (b = 0.195, p < 0.001), Opportunity (b = 0.165, p < 0.001), and Fashion sense (b = 0.441, p < 0.001) significantly affect Motivation. Fashion sense (b = 0.581, p < 0.001) significantly influences Opportunity.

**Table 6:** Path analysis table.

DV	IV	Regression weight	SE	Z-value	Value of p	Standardized path coefficient
Behavior (R^2^ = 0.361)	Capability	0.208	0.082	2.547	0.011	0.157
	Motivation	0.181	0.084	2.148	0.032	0.135
	Opportunity	0.530	0.078	6.833	0.000	0.413
Capability (R^2^ = 0.422)	Fashion sense	0.554	0.042	13.271	0.000	0.650
Motivation (R^2^ = 0.618)	Capability	0.195	0.049	3.985	0.000	0.196
	Opportunity	0.165	0.047	3.548	0.000	0.172
	Fashion sense	0.441	0.048	9.245	0.000	0.520
Opportunity (R^2^ = 0.433)	Fashion sense	0.581	0.044	13.120	0.000	0.658

**Figure 4:**
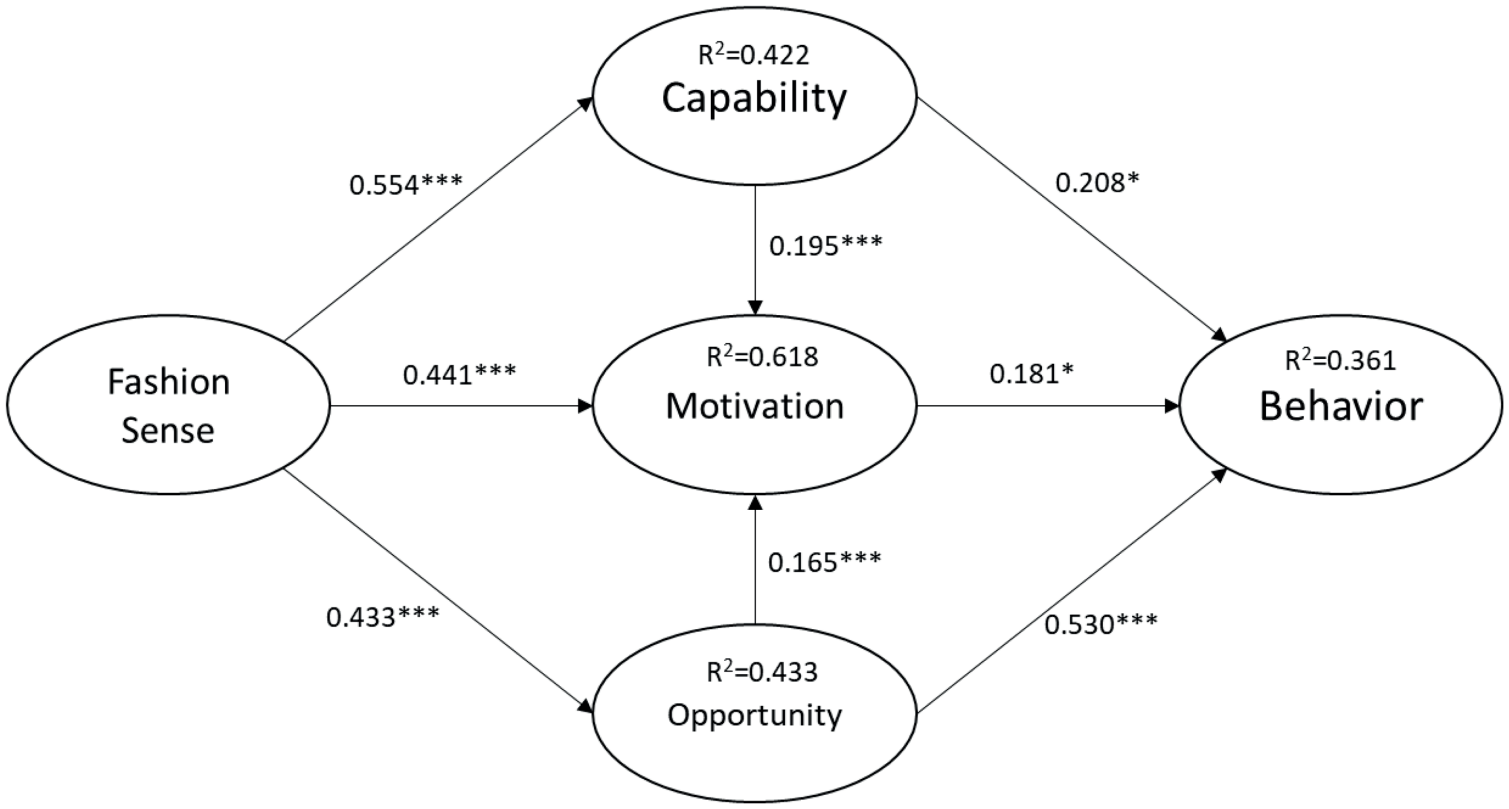
Structural model results.

These results support the research questions for this model. The explanatory power of Capability, Motivation, and Opportunity in terms of explaining Behavior is 36.1%. The explanatory power of Fashion sense to terms of explaining Capability is 42.2%. The explanatory power of Capability, Opportunity and Fashion sense in terms of explaining Motivation is 61.8%. The explanatory power of Fashion sense in terms of explaining Opportunity is 43.3%. Figure 4 shows the results for the structural model for standardized path coefficients, significant level and R-square.

## Discussion and conclusion

This study determines the factors that influence the usage behavior of beauty industry matchmaking platform members during the COVID-19 pandemic. The COM-B model framework is used to determine the factors for the pursuit of beauty by users of media use platforms. The COM-B model is verified using fashion sense as a construct. A novel research framework and related hypotheses are proposed. Data was collected using a questionnaire and the structural equation model is used to test the hypotheses.

### Academic contributions

COVID-19 has caused public health and virus transmission crises in various countries and regions around the world so it has a significant impact on livelihoods and economies. The impact of the epidemic is directly reflected in changes in the consumer market, which is dealing with new consumption patterns and habits in an economy that is contactless. By promoting digital applications, the beauty industry has been able to maintain communication with consumers because during a pandemic, the BIMP appointment app could be used with an acceptable motivation at the psychological level to overcome psychological barriers ife (Oliveira et al., 2021). It is also a source of stress relief and increases self-confidence. Beauty consumers have become used to booking online or in-home services using the APP and the online booking mechanism is increasing in importance. For members who use the APP service platform in the pursuit of beauty, this study determines their motive for doing so.

This study uses the COM-model behavior research framework. The fashion sense construct is used to verify the influences on behavioral change and to determine the correlation between fashion sense and the ability, motivation and opportunity to influence behavior. Table 7 presents the results for validation of the study hypotheses with the standardized regression coefficient, z-value, and value of p.

**Table 7:** Hypothesis testing result.

Hypothesis	Unstandardized path coefficient	Z-value	Value of p	Standardized path coefficient
H1: Fashion sense → Capability	0.554	13.271	0.000	0.650
H2: Fashion sense → Motivation	0.441	9.245	0.000	0.520
H3: Fashion sense → Opportunity	0.581	13.120	0.000	0.658
H4: Capability → Motivation	0.195	3.985	0.000	0.196
H5: Opportunity → Motivation	0.165	3.548	0.000	0.172
H6: Capability → Behavior	0.208	2.547	0.011	0.157
H7: Motivation → Behavior	0.181	2.148	0.032	0.135
H8: Opportunity → Behavior	0.530	6.833	0.000	0.413

#### The effect of BIMP users’ fashion sense on the beauty care capability

The results of this study show that BIMP users’ sense of fashion has a significant positive effect on beauty care capability. The greater the sense of fashion, the greater is the beauty care capability of the user. The greater the sense of fashion, the greater is the sensitivity to beauty and fashion information and the greater is the knowledge of beauty.

The highest score of 6.21 is for “My friends and family think I should take preventive measures when I go to a beauty technician.” This result is consistent with those of previous studies (Farooq, 2020) so individuals remind each other of the health risks of the COVID-19 pandemic. It also shows that precautionary measures are already prevalent in the minds of many individuals.

The statement, “I know the common sense of protection during beauty care,” has the lowest, score of 5.64. Beauty platform users maintained a sense of fashion during the epidemic and have general knowledge of protection, so there was concern about the capability to protect against viral infection.

#### The influence of BIMP users’ fashion sense on the motivation for self-seeking beauty

The results of this study show that BIMP users’ sense of style has a significant positive effect on self-motivation to pursue beauty. This result is the same as the results for previous studies (Trekels and Eggermont, 2017). The statement, “I feel very confident after a beauty care,” has the highest score of 6.14. The greater the sense of style, the greater is the motivation to pursue beauty and the greater is the confidence. The results of study also show that there is little difference between the scores for the five questions that pertain to the motivational component of the questionnaire, so beauty and grooming as a daily activity is very closely related to pleasure, happiness and interpersonal relationships and self-confidence.

#### The impact of BIMP users’ fashion sense on future collaboration opportunities

The results of this study show that BIMP users’ fashion sense has a significant positive impact on future cooperation opportunities. The unstandardized regression analysis coefficient of 0.581 indicates that the path hypothesis also has a significant impact. This shows that the greater the user’s sense of fashion, the more interested are platform members in future collaboration opportunities.

The highest score is 5.72 for “attracting potential consumers through the BIMP.” This result clearly shows that the app operates well as a three-party appointment service and the platform members are very positive. For this study, 74.1% of users aged 21 to 50 years old use BIMPs. This is similar to value that is defined by the GWI (2020) survey, which shows that 58% of individuals aged 16 to 58 years used social media during the COVID-19 pandemic.

This shows that the consumer market has shifted from physical stores to contactless business opportunities. Therefore, products with a sense of fashion, reputation and partnerships that create visibility are attractive to r BIMP users in the virtual space of application software.

#### Validating COM-B theory behavioral influences

In terms of the research context of the BIMP, this study adds fashion sense as an antecedent variable to the COM-B research framework to determine the extent to which fashion sense affects capabilities, motivations and opportunities. In terms of the path analysis for behavior that influences capability, motivation and opportunity, the standardized regression analysis coefficients are: capability (β = 0.157, p = 0.011), motivation (β = 0.135, p = 0.032) and opportunity (β = 0.413, p = 0.000). The results of this study confirm the hypothesis that capability, motivation and opportunity have a highly significant effect on behavior, which is consistent with the theoretical framework of the COM-B model.

Opportunity is the factor that most influences behavior, so users on the BIMP have the greatest demand for future cooperation opportunities to improve their reputation. The behavioral component question, “I often show my hair with other members on BIMPs,” has the lowest score, possibly because the platform uses members to show their value, or the platform does not provide enough incentives to promote the value of the app. Sharing, communication and discussion form the basis of the platform’s operation.

In terms of the path analysis for fashion sense influence capability, the standardized regression analysis coefficient is 0.650 (p = 0.000), which shows that fashion sense significant influences capability. The standardized regression analysis coefficients for the path analysis for capability, opportunity and sense of fashion influencing motivation are: capability (β = 0.196, p = 0.000), opportunity (β = 0.172, p = 0.000) and sense of fashion (β = 0.520, p = 0.000). The results of this study verified that capability, opportunity, and fashion ability significantly influence motivation.

The explanatory power of capability, motivation, and opportunity for behavior is 36.1%. The explanatory power of fashion sense for capability is 42.2%. The explanatory power of capability, motivation and fashion sense for motivation is 61.8%. The explanatory power of fashion sense for opportunity is 43.3%. The results of this study demonstrate that the influences that are determined by the COM-B research framework are confirmed.

### Practical suggestions

COVID-19 has threatened people and economies since 2020 onward so cities around the world have taken measures to prevent the spread of the virus. Most beauty technicians serve consumers through direct contact in exchange for payment. During a pandemic, city lockdowns and interpersonal restrictions have threatened the survival of beauty technicians, so the “BIMP three-party appointment platform” is an effective method of ensuring consumption and increasing employment opportunities. An understanding of customers’ lives and needs is required to promote the most suitable products and services to people whom they benefit (Keller and Kotler, 2012).

Social media and applications will be more common in the future in response to the new consumption habits of consumers. This study validates the capability, motivation, and opportunity to influence consumer behavior in the beauty industry by adding a fashion sense construct to the COM-B model. The results show that the pandemic does not affect motivation to pursue beauty. Most consumers are aware of measures to control the pandemic and wish to collaborate with other users of the platform on BIMP. Each component is closely related to platform usage behavior. The results of this study show that the Beauty Industry three-party appointment service platform promotes service opportunities in terms of capacity, motivation, and opportunity to influence the behavior of platform members.

The results of this study show that the use of the platform increases the function of the epidemic prevention interface. A study of BIMP Users’ Beauty Care Capability shows that consumers, stores and beauty technician members place great importance on preventive measures. Practical protection measures be added so that consumers can communicate with each other online when booking services. A declaration of current physical status, vaccination status and the environment in which the service is operated allows the consumer, the store and the beauty technician to interact physically and increase mutual trust. Viruses mutate rapidly so information about virus prevention must be updated frequently.

The motivation to use the platform to pursue beauty and initiate positive mindfulness should be increased self-confidence is increased after a beauty treatment and inspirational short stories would be a useful addition to the member platform. Beauty industry uses platform members are mainly women so society generally recognizes that women are professionally disadvantaged. The new post-epidemic era is driven by women’s confidence in the pursuit of beauty.

The results of this study also show that fashion sense has a positive and significant effect on the motivation to pursue beauty. Therefore, fashion information, such as popular hair styles, beauty trends or fashion reports about entertainment celebrities could be added to the BIMP to enhance members’ perception of fashion and enhance the motivation to pursue beauty.

In terms of opportunities for future cooperation in promoting the use of BIMP, most members who use the application software do so in the pursuit of beauty and others are encouraged to do likewise so the current Chinese interface can be used in Taiwan for experimental consumer testing and then extended to the Chinese community, including the 1.7 billion population on mainland China.

### Research limitations and future work

This study determines the influence of the beauty industry’s three-party platform on users’ behavioral changes using COM-B as the basis of the theoretical model and fashion sense as the antecedent variable. Due to time and funding constraints, this study only validates the current five constructs. Future studies could add new constructs, such as service convenience and platform visibility. This study only concerns the behavior of BIMP members on the platform so future experimental studies might determine consumer attributes, such as satisfaction with and loyalty to the BIMP. Results might also be qualitatively analyzed using focus group interviews and in-depth interviews.

## Data availability statement

The raw data supporting the conclusions of this article will be made available by the authors, without undue reservation.

## Author contributions

Y-WC conceived of the presented idea, developed the theory, and performed the computations. YH verified the analytical methods. Both authors discussed the results and contributed to the final manuscript.

## Conflict of interest

The authors declare that the research was conducted in the absence of any commercial or financial relationships that could be construed as a potential conflict of interest.

## Publisher’s note

All claims expressed in this article are solely those of the authors and do not necessarily represent those of their affiliated organizations, or those of the publisher, the editors and the reviewers. Any product that may be evaluated in this article, or claim that may be made by its manufacturer, is not guaranteed or endorsed by the publisher.
